# ParaHox Genes Revisited: From Gut Patterning to Integrated Axial and Neural Organization in Rotifera

**DOI:** 10.1002/jez.b.70019

**Published:** 2026-03-15

**Authors:** Andreas C. Fröbius, David B. Mark Welch, Holger Herlyn

**Affiliations:** ^1^ Institute for Veterinary Physiology and Biochemistry, Faculty of Veterinary Medicine Justus Liebig University Giessen Giessen Germany; ^2^ Josephine Bay Paul Center for Comparative Molecular Biology and Evolution Marine Biological Laboratory Woods Hole Massachusetts USA; ^3^ Institute of Organismic and Molecular Evolution (iomE), Anthropology Johannes Gutenberg University Mainz Mainz Germany

**Keywords:** Acanthocephala, *Brachionus*, *Cdx*, gnathifera, *Gsx*, *ParaHox*

## Abstract

The *ParaHox* homeobox genes *Gsx*, *Xlox*, and *Cdx* are evolutionarily related to *Hox* genes and form part of the ANTP‐class homeobox gene repertoire. Comparative genomic data indicate that *ParaHox* genes were already present before the cnidarian‐bilaterian split. Across metazoans, *ParaHox* genes often show conserved associations with the anteroposterior body axis and have been implicated in both gut patterning and neural development, although gene complements and genomic organization vary substantially among lineages. To investigate *ParaHox* gene deployment in Gnathifera, we identified orthologs of *Gsx* and *Cdx* across gnathiferan lineages, including Chaetognatha, Monogononta, Bdelloidea, Seisonidea, and Acanthocephala, but found no evidence of *Xlox*, indicating a reduced *ParaHox* gene complement. We analyzed the genomic organization and embryonic expression of *Gsx* and *Cdx* in the monogonont rotifer *Brachionus manjavacas* (*Bm*). Genomic mapping revealed a dispersed *ParaHox* configuration, with *Bm‐Gsx* and *Bm‐Cdx* separated by 4.4 Mb. Using whole‐mount *in situ* hybridization, we detected *Bm‐Gsx* expression in neurons of the foot region, as well as in a small number of cells with neuronal characteristics and probable involvement in stomatogastric system development. *Bm‐Cdx* was expressed in FMRFamide‐positive cells associated with the bladder, consistent with a neuroepithelial identity. Together, these data indicate that in rotifers, *ParaHox* gene expression is predominantly associated with neural structures. We propose that this pattern represents a derived condition reflecting the compact body plan and reduced gut organization characteristic of rotifers, highlighting the evolutionary flexibility of *ParaHox* gene deployment under lineage‐specific developmental constraints.

## Graphical abstract

In rotifers, *ParaHox* genes show a dispersed genomic organization, with *Xlox* absent across gnathiferans. Exclusive neuronal expression of *Gsx* and *Cdx* reveals that ancestral *ParaHox* genes coordinated neural and epithelial development beyond gut patterning, suggesting an integrated role in early bilaterian body plan organization.

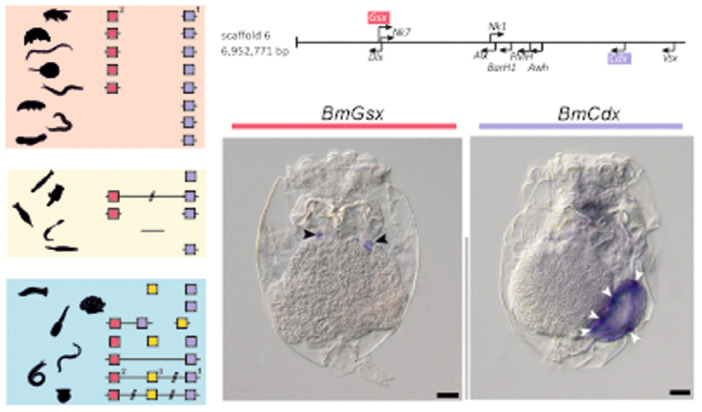

## 1 Introduction

Fossils about 530 million years old illustrate fast phenotypic innovation and diversification of the Metazoa. This so‐called Cambrian explosion led to the abrupt appearance of many metazoan body plans from which extant fauna evolved (Zhang and Shu 2021). The genetic basis of this early radiation is a matter of intense research, largely focusing on genes predating this event (Brooke et al. 1998). This is especially the case with respect to gene clusters that are involved in the ontogenetic patterning of body regions by modulation of developmental cascades. Among the key effectors are two families of antennapedia (ANTP) class homeodomain transcription factors encoded by *Hox* and *ParaHox* genes (Brooke et al. 1998; Garcia‐Fernàndez 2005). The first are mostly organized in genomic clusters with staggered expression domains in the embryo, reflecting cluster structure (spatial collinearity). The encoded *Hox* transcription factors imprint regional identities along the anteroposterior axis of developing animals and pattern different parts of the nervous systems (Carroll 1995; Fröbius et al. 2008; Krumlauf 1994). Correspondingly, *Hox* gene number, cluster structure and expression have been directly associated with the evolution of metazoan body plans (Gellon and McGinnis 1998). The three members of the *ParaHox* gene family were originally discovered in the cephalochordate *Branchiostoma floridae* (Brooke et al. 1998). Lack of awareness of orthology relations resulted in redundant naming of the genes across different animal phyla as *Gsx/Gsh/Ind*, *Pdx/Xlox*, and *Cdx/Cad*. These genes are homologous to anterior *Hox* genes, *Hox3* genes, and posterior *Hox* genes, respectively. Important characteristics for the classification of homeodomain genes, including members of the *ParaHox* family, rely on the presence of conserved diagnostic amino acid motifs within and flanking the homeodomain of the encoded proteins (de Rosa et al. 1999). Most protostomian *Gsx/Ind* genes analysed have a cysteine‐rich region downstream of the homeodomain, a feature lacking in diploblasts, urochordates and vertebrates. The proteins encoded by *Cdx/Cad* include a hexapeptide motif at varying distances upstream of the homeodomain, which is also present in some *Hox* proteins. This motif is less widespread across animals and might function in concert with cofactor proteins (Neuteboom et al. 1995).

**Figure 1:**
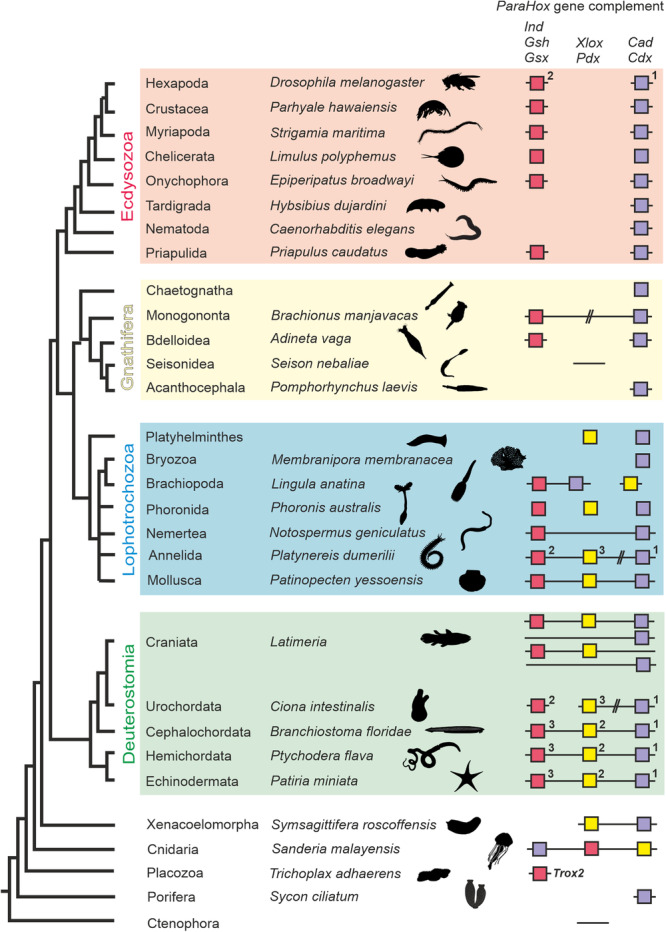
*ParaHox* gene repertoires across Metazoa. The tree on the left displays bilaterian phylogeny with Ctenophora as an outgroup, based on the Gnathiferan phylogeny of Lewin and Luo 2025 and Vasilikopoulos et al. 2024. Color‐coded boxes depict *ParaHox* genes present in representative species. Underlying lines indicate known genomic organisation. Clustered genes are connected. Hashes indicate large distances between genes on the same genomic scaffolds. 1, 2, 3: temporal order of gene activation during embryogenesis, if known and not initiated simultaneously.

The high similarity between *ParaHox* and *Hox* homeodomains, along with the finding that both gene families are often organized in genomic clusters, suggests their common origin from a proto‐*Hox* cluster consisting of 2‐4 genes in early metazoan evolution (Brooke et al. 1998; Garcia‐Fernàndez 2005). The probable metazoan origin, coupled with the collinear patterning of *Hox* gene expression along the anteroposterior body axis and early expression of *Xlox* in the midgut and *Cdx* in the hindgut of the *B. floridae*, fostered the hypothesis that *ParaHox* genes may have patterned the digestive tract in the last common bilaterian ancestor. In support of this view, *ParaHox* and *Hox* genes are lacking in Ctenophora (Ryan et al. 2010). While some studies failed to identify *ParaHox* genes in sponges (Pastrana et al. 2019), other analyses reported a *Cdx*‐like gene in calcisponges (Fortunato et al. 2014), indicating that the presence of *ParaHox* genes in Porifera remains unresolved. Recent genomic analyses of cnidarians have revealed that *ParaHox* gene complements and their genomic organization are more diverse than previously appreciated. Several cnidarian species possess all three *ParaHox* genes, *Gsx*, *Xlox*, and *Cdx*, arranged in a genomic cluster (Figure 1) (Law et al. 2023; Nong et al. 2020). These findings suggest that *ParaHox* genes and their association in genomic clusters predate the cnidarian‐bilaterian split, while also highlighting early flexibility in gene content and gene order. The evolutionary depth of *ParaHox* genes is further underscored by the presence of a *ParaHox*‐related gene with high similarity to *Gsx* (*Trox‐2*) in Placozoa (Figure 1) (Jakob et al. 2004).

The phylogenetic position of Xenacoelomorpha, consisting of acoel flatworms and the genus Xenoturbella, remains debated. While some analyses place this lineage as the sister group to all other bilaterians, alternative hypotheses recover Xenacoelomorpha within Deuterostomia as sister to Ambulacraria. *Gsx* and *Cdx* genes have been isolated from *Xenoturbella bocki* and various acoel species (Brauchle et al. 2018), and *Xlox* and *Cdx* from *Nemertoderma westbladi* (Figure 1) (Jimenez‐Guri et al. 2006). However, the genomic organisation of these *ParaHox* members in xenacoelomorphs is currently unknown.

All three *ParaHox* genes have been identified in deuterostome lineages, both in clusters and as dispersed genes (Figure 1) (Arnone et al. 2006; Brooke et al. 1998; Ikuta et al. 2009). As in the cephalochordate genus *Branchiostoma* mentioned above, species of Echinodermata and Hemichordata possess a single complete cluster of three *ParaHox* genes (Annunziata et al. 2013; Ikuta et al. 2013). In the urochordate *Ciona intestinalis*, the three genes are dispersed on two chromosomes, with *Xlox* and *Cdx* residing about 240 kb apart on chromosome arm 14q and *Gsx* localizing to 2q (Shoguchi et al. 2006). Repeated genome duplication events early in the vertebrate stem lineage led to the formation of multiple *ParaHox* loci in vertebrates, with four *ParaHox* clusters on four chromosomes representing a typical condition in some lineages (e.g. mammals), and with varying subsets of the three *ParaHox* genes retained in different Craniata species (Figure 1) (Ferrier et al. 2005; Mulley et al. 2006; Mulley and Holland 2010, 2014).

Within Protostomia, the structure of the *ParaHox* cluster is less strongly conserved, with different patterns in the three major protostome clades: Ecdysozoa, Platytrochozoa, and Gnathifera (Figure 1). The central *ParaHox* gene, *Xlox/Pdx*, might have been lost in the ecdysozoan stem line, as it has not yet been recovered from any ecdysozoan genome. The remaining genes do not establish clusters and, instead, occupy remote genomic loci in Onychophora (search of the genome of *Epiperipatus broadwayi*; assembly ASM2802345v1), Myriapoda (Chipman et al. 2014), Chelicerata (Nong et al. 2020), Crustacea (Kao et al. 2016), and Insecta (Macdonald and Struhl 1986; Weiss et al. 1998). Within Ecdysozoa, the *Gsx/Gsh* ortholog has not been identified in Nematoda (Ruvkun and Hobert 1998) and Tardigrada (Boothby et al. 2015; Smith et al. 2016), consistent with lineage‐specific loss in these groups. In contrast, *Gsx* is present in Priapulida, as supported by available sequence data and our phylogenetic analyses. Thus, Priapulida retains at least two *ParaHox* genes, *Gsx* and *Cdx/Cad*. Within Lophotrochozoa, orthologs of all three *ParaHox* genes have been isolated from various taxa (Figure 1): Annelida (Fröbius and Seaver 2006; Hui et al. 2009; Park et al. 2006), Sipunculida (Ferrier and Holland 2001), Mollusca (Biscotti et al. 2014; Fritsch et al. 2016; Wang et al. 2017), Brachiopoda (Luo et al. 2015), and Phoronida (Luo et al. 2018). Genomic organisation varies however, and a complete cluster with all three genes in close vicinity to each other has only been found in the bivalve *Patinopecten yessoensis* (Wang et al. 2017). In the nemertean *Notospermus geniculatus* the central *Xlox* gene is missing, leaving *Gsx* and *Cdx* orthologs to form an incomplete cluster (Luo et al. 2018). Furthermore, Bryozoa seem even to have lost *Gsx* and *Xlox* orthologs (Saadi et al. 2023). Within Platyhelminthes, the presence of either *Xlox* and *Cdx* genes or a single *Xlox* gene have been reported from the polyclad *Discocelis tigrina* (Saló et al. 2001) and the triclad *Schmidtea mediterranea* (Martín‐Durán and Romero 2011), respectively. Compared to these protostome lineages, little is known about the complement of *ParaHox* genes and their genomic organization and developmental relevance in the sister group of Platytrochozoa, Gnathifera. This group of helminths includes Chaetognatha, Micrognathozoa, Gnathostomulida, and Rotifera or Syndermata (Monogononta, Bdelloidea, Seisonidea, and Acanthocephala), whereby each taxon can be considered as a sister group to a clade comprised of the subsequent taxa (Ahlrichs 1997; Haffner 1950; Herlyn 2021; Lewin and Luo 2025; Mark Welch 2000; Marlétaz et al. 2019; Rieger and Tyler 1995; Sielaff et al. 2016; Vasilikopoulos et al. 2024)

Here we provide insight into the complement and genomic organization of *ParaHox* genes in Gnathifera, describe the spatiotemporal expression of *ParaHox* genes in the monogonont rotifer *Brachionus manjavacas* as revealed by whole mount in‐situ hybridization from embryonic stages to adults, and discuss these results in the context of previously published data on *ParaHox* genes in animals. As with other rotifers, *B. manjavacas* is eutelic; after an initial phase of cell division and complete morphogenesis of the body plan, cells or syncytia change only in size. In filter‐feeding rotifers, the size difference between neonates and adults is based only on water uptake and successive expansion (Fontaneto and Melone 2005). Remains of late gene expression during embryogenesis can often still be detected in young adults (Fröbius and Funch 2017). Except for the nervous system, comprising the majority of the cells of a rotifer, most tissues consist of only a small number of cells or syncytia and often have a rather simple composition (Clément and Wurdak 1991). These features and a transparent body allow straightforward analyses of gene expression in this gnathiferan model organism. In contrast to earlier hypotheses emphasizing a primary role of *ParaHox* genes in gut patterning, our findings add to a growing body of comparative evidence indicating that *ParaHox* genes are widely involved in nervous system patterning across metazoans.

## 2 Methods and Materials

### 2.1 *Brachionus manjavacas* RUS Culture

Resting eggs of the *B. manjavacas* strain RUS were originally obtained from a commercial supplier (Florida Aqua Farms). A culture of *B. manjavacas* RUS was maintained, and embryos and adults of amictic females were collected, fixed and permeabilised as previously described (Fröbius and Funch 2017).

### 2.2 Cloning of *Brachionus manjavacas ParaHox* Genes

We used the RNeasy kit (Qiagen) for RNA extraction from mixed developmental stages of amictic embryos of *B. manjavacas* RUS. Rapid amplification of 5′ and 3′ cDNA ends (RACE) relied on the SMARTerRACE kit (Takara Clontech). Initially, fragments of the homeodomains of *B. manjavacas Bm‐Gsx* and *Bm‐Cdx* were isolated using the degenerate primers NSNSRRM (5′‐AAYTCMAACTCGMGGMGAATG‐3‘) and SNEKCKC (5′‐CAYTTGCAYTTYTCATTYGAC‐3‘) as well as KTRTRDKY (5′‐AARACNMGNACNMGNGAYAARTA‐3′) and NRRAKERK (5′‐TTNCKYTCYTTNGCNCKNCKRTT‐3′), respectively, with a 1:1 mixture of both RACE cDNAs as template. We recovered 5′ and 3′ ends of the *Bm‐Gsx* and *Bm‐Cdx* transcripts by RACE with gene‐specific primers according to the manufacturer′s instructions. All fragments isolated were cloned into the pGEM‐Teasy vector system (Promega) and sequenced by Seqlab‐Microsynth (Göttingen, Germany). Sequences were submitted to GenBank at NIH under accession numbers PV294966 for *Bm‐Gsx* and PV294965 for *Bm‐Cdx*.

### 2.3 Riboprobe Synthesis and Whole‐Mount In‐Situ Hybridization

Templates for riboprobe synthesis were generated from clones containing a 482 bp 5′ RACE fragment of *Bm‐Gsx* and a 944 bp 5′ RACE fragment of *Bm‐Cdx* by polymerase chain reaction (PCR) with primers corresponding to the SP6 and T7 promotor regions of the vector. A labelled riboprobe was synthesized in vitro using the MEGAscript High Yield Transcription Kit (Invitrogen, Fisher Scientific) incorporating digoxigenin‐11‐UTP (Roche). Whole‐mount in situ hybridization was performed as previously described (Fröbius and Funch 2017). Both riboprobes were used at a working concentration of 3 ng/µl. Results were analysed using differential interference contrast optics on an Olympus BX51 microscope. Photomicrographs were taken with an Olympus DP27 camera. A detailed protocol is available upon request.

### 2.4 Linkage Analysis

After initial isolation of the *ParaHox* sequences by PCR, access to a newly generated draft assembly of the *B. manjavacas* RUS genome became available, allowing us to analyze the positions of the *ParaHox* genes and their possible linkage in the genome. This genome project was deposited at DDBJ/ENA/GenBank under the accession JBTYIN000000000. The version used in this paper is version JBTYIN010000000. The Basic Local Alignment Search Tool (BLAST) version +2.10.1, obtained from NCBI (https://blast.ncbi.nlm.nih.gov/blast/Blast.cgi), was used to identify the genomic scaffold containing *B. manjavacas ParaHox* genes and to determine the structure of their transcriptional units (intron/exon structure) by comparison with the previously isolated nucleotide sequences of *Bm‐Gsx* and *Bm‐Cdx* mRNA. In addition, scaffold data were analysed using the gene prediction algorithm Genscan (http://hollywood.mit.edu/GENSCAN.html) and by tblastn searches querying amino acid sequences of various homeoboxes belonging to different homeodomain gene classes. To assess the robustness of gene content and local gene order, scaffold organisation was independently cross‐validated using an additional publicly available *B. manjavacas* genome assembly deposited at NCBI (accession number ASM1905480), which revealed identical identity and relative arrangement of the homeobox genes analyzed.

### 2.5 Phylogenetic Analysis

The cloned sequences were initially identified by BLASTX searches of the GenBank database at NCBI. Amino acid sequences of *Gsx/Gsh/Ind* genes and *Gsx/Cad* genes, as well as *Hox1* genes and posterior *Hox* genes (PG9‐14, *AbdB*, *Post1*, *Post2*, *PostA* and *PostB*) of representatives of various animal taxa were first aligned separately with ClustalX2.1. Species and accession numbers of all sequences included in the phylogenetic analyses are provided in Supporting Information Table S1. Sequences were manually pruned of ambiguous sites between what corresponds to the hexapeptide region and some conserved amino acids upstream of the homeodomain of *Cdx* and *Hox1* proteins. We did the same for ambiguous bases in gene sections coding for protein stretches between the homeodomain and cysteine‐rich region of *Gsx* proteins. Hexapeptide motifs and cysteine‐rich regions were included in orthology analyses of translated sequences in addition to the highly conserved 60 aa homeodomain. In detail, we ran MAFFT on the translated sequences and then merged the three alignments. Prottest 3.4.2 determined the JTT + G + I substitution model to be best suited for phylogenetic analyses to be run on the Cipres Science Gateway 3.3 web portal. Bayesian phylogenetic inference was performed in BEAST2 using an MCMC chain of 40 million generations, sampling every 1000 generations. Convergence and effective sample sizes were assessed in Tracer, and the first 10% of samples were discarded as burn‐in. The target tree resulting from the BEAST2 analysis was generated with TreeAnnotator using the maximum clade credibility option. Maximum Likelihood (ML) analysis by RAxML‐NG was performed with 1000 bootstrap replicates, and transfer bootstrap expectation (TBE) values were calculated. Resulting phylograms were rooted in FigTree 1.4.4 (https://github.com/rambaut/figtree/releases). The same phylogenetic workflow was applied to infer orthology relationships of additional homeobox genes located on the *ParaHox*‐containing scaffold (Supporting Information Figure S1).

## 3 Results

Initial PCR amplification of partial *Bm‐Gsx* and ‐*Cdx* genes was carried out on mixed embryonic and adult cDNA as template. We used degenerate primers binding to conserved regions coding for homeodomains to recover fragments of putative orthologs. Additional 3′‐ and 5′‐sequence information was recovered by RACE PCR. Corresponding Sanger sequences were matched with Genbank entries by blastx (http://www.ncbi.nlm.nih.gov/BLAST). No *Xlox* orthologue could be identified in *B. manjavacas* despite the use of different sets of degenerate primers. In addition, similarity searches using *Xlox/Pdx* homeodomains from diverse lophotrochozoan and deuterostomian species as queries against the *B. manjavacas* genome assembly failed to recover an *Xlox* orthologue. Comparable searches in additional publicly available gnathiferan genome assemblies yielded the same result (Figure 1).

### 3.1 Orthology Assignment of *B. manjavacas ParaHox* Genes

**Figure 2:**
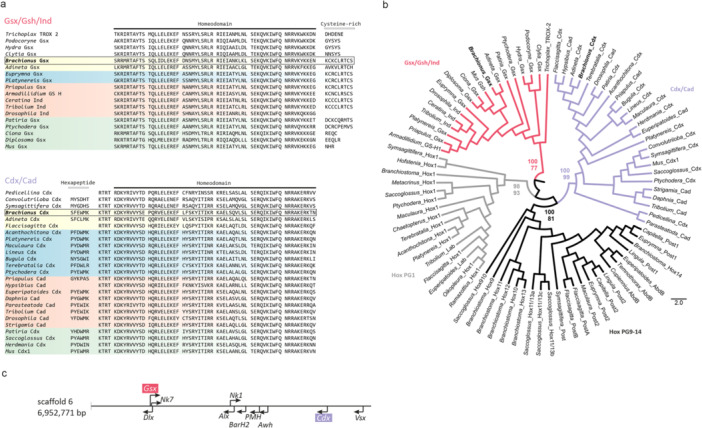
Representatives of *Gsx/Gsh/Ind* and of *Cdx/Cad* gene families are present in the genome of *Brachionus manjavacas*. (a) Amino acid alignment of the homeodomain and flanking regions in *ParaHox* transcription factors across the animal kingdom. Color coding: white: diploblastic metazoans; yellow: Gnathifera; blue: Lophotrochozoa; red: Ecdysozoa; green: Deuterostomia. Most protostomian *Gsx* and *Ind* genes analysed feature a cysteine‐rich region 3′ of the homeodomain also present in *Bm‐Gsx* but missing in diploblasts and higher deuterostomes. The protein sequence of *Cdx/Cad* genes contains a hexapeptide motif at varying distances 5′ to the homeodomain, also present in some *Hox* genes. This motif, thought to function in concert with cofactor proteins, exhibits higher variation across Metazoa than the rest of the gene. These motifs have been included in orthology analyses in addition to the highly conserved 60‐aa homeodomain. (b) Phylogenetic tree depicting orthology assignments for both genes isolated from *B. manjavacas*. Both maximum likelihood (ML) (1st value) and Bayesian analysis (2nd value), strongly support the orthology assignments of *Bm‐Gsx* and *Bm‐Cdx* in their respective gene families. *Hox1* genes (closely related to *Gsx*) and posterior *Hox* genes (closely related to *Cdx*) were used as outgroups. (c) The *ParaHox* cluster of *B. manjavacas* is dispersed. Genomic linkage of *Bm‐Gsx* and *Bm‐Cdx*. Both *ParaHox* genes of *B. manjavacas* are located on scaffold 6, spanning 6.952.771 bp, however, the genes are separated by 4.425 Mb and located on opposite strands. The ORFs of several other genes were identified between them. The schematic shows the position of other ANTP class genes.

The presence of conserved diagnostic amino acid motifs within and flanking the homeodomain are important characteristics for the classification of homeodomain genes. In the present study, we included the cysteine‐rich region and hexapeptide motifs in orthology analyses of translated sequences, in addition to the highly conserved 60 aa homeodomain (Figure 2a). Orthology assignment for both genes was determined by multiple methods. Both Maximum Likelihood (ML) and Bayesian Inference (BI) strongly supported the orthology assignments of *Bm‐Gsx* in the *Gsx/Gsh/Ind*‐ and of *Bm‐Cdx* in the *Cdx/Cad* gene families (Figure 2b). We did not identify a representative of the *Xlox/Pdx* family in the published genome assembly of *B. manjavacas* as mentioned above, nor in the nuclear genome assemblies of other members of Gnathifera (Figure 1).

### 3.2 Genomic Linkage

Both *ParaHox* genes in *B. manjavacas* RUS localized to a genome assembly scaffold spanning 6.952.771 bp. However, the two genes are separated by a substantial genomic distance and do not form a tight *ParaHox* cluster. In fact, the single‐exon *Bm‐Gsx* gene (630 bp) located towards the 5′‐end of the scaffold, while the *Bm‐Cdx* located 4.425 Mb 3′ of *Bm‐Gsx* (Figure 2c). The three exons of *Bm‐Cdx* (724, 146, and 579 bp) were separated by small introns (284 bp and 56 bp), resulting in a transcription unit of 1789 bp. The genes resided on opposite strands, and open reading frames (ORFs) of several other genes extended between them. Among the latter were ANTP class genes *Dlx*, *BarH1*, *Awh*, *Alx*, *Nk1*, and *Nk7* (Figure 2c).

### 3.3 *ParaHox* Gene Expression in *B. manjavacas*

**Figure 3:**
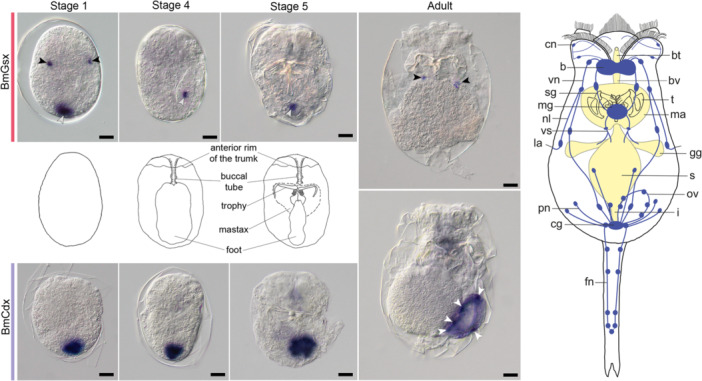
Expression of *ParaHox* genes during embryogenesis of *Brachionus manjavacas*. Whole‐mount in situ hybridization on amictic female embryos and adults. Anterior is to the top. Ventral or slightly ventrolateral views are shown. Schematics of the embryonic stages are depicted in the middle. Expression domains marked: black arrowheads: *Bm‐Gsx* expression in the trunk region, part of the stomatogastric nervous system; grey arrowheads: posterior expression domain/serotonergic neurons in the foot; white arrowheads: *Bm‐Cdx* expression in pericarya of FMRF‐positive neurons forming the wall of the osmotic vesicle. Weak staining occasionally observed in the region of the buccal velum is interpreted as non‐specific background, as a similar signal is detected with probes against unrelated genes. Simplified diagram of the nervous system (blue) and digestive tract (yellow) of *Brachionus manjavacas*. Nerves on the dorsal side and scalar nerves are not shown. b, brain; bt, buccal tube; bv, buccal velum; cg, caudal ganglion; cn, corona nerve; fn, foot nerve; gg, gastric gland; i, intestine; l, lateral antenna; ma, mastax; mg, mastax ganglion; nl, nerve to lateral antenna; ov, osmotic vesicle; pn, pedal nerves; s, stomach; sg, stomatogastric nerve; t, trophi; vn, ventral nerve; vs, visceral nerve. Scale bar, 10 µm.

Whole‐mount *in situ* hybridization (Figure 3) revealed initiation of *Bm‐Gsx* expression after the completion of cleavage in amictic embryos of *B. manjavacas*. With the onset of morphogenesis, we found *Bm‐Gsx* to be first expressed in a small domain of 1–2 ectodermal cells ventrally at the posterior embryonic pole. At morphogenesis stage 1, *Bm‐Gsx* expression signal emerged in two single ectodermal cells in the anterior half of the embryo, especially, ventrolaterally of the future trunk. These cells were no longer detectable from stage 2 on, indicating merely transient expression. In contrast, we noticed the posterior expression domain to persist throughout the following stages, thereby shifting anteriorly along the ventral midline. At stage 4, the expression domain localized to two FMRFamide positive neurons connecting the foot to the caudal ganglion (see [Fröbius and Funch 2017], Supporting Information Figure 6f,g for reference). Upon hatching, neuronal expression in the foot was downregulated and no longer detectable in adult amictic females. At the same time, a weak signal was emitted from small clusters of cells with neuronal characteristics in the region between mastax and stomach, may be part of the stomatogastric nervous system in *B. manjavacas*.

Transcripts of the *caudal/Cdx* homolog of *B. manjavacas* were not detected in cleavage stages. Upon the onset of morphogenesis, expression of *Bm‐Cdx* is set on in a distinct ectodermal expression domain at the posterior pole of the embryo. Initially being restricted to this area, the pattern shifted rostrally and dorsally in further development. On its way, the domain expressing *Bm‐Cdx* first expanded and then dissociated into several smaller expression domains. Subsequently, the cells exhibiting *Bm‐Cdx* expression were relocated to the dorsal left side of the embryo. By the end of morphogenesis, the *Bm‐Cdx*‐expressing cells formed a ring‐like structure located at the dorsal left side of the posterior half of the trunk, just below the body wall of the young rotifer. The position of the structure coincided with FMRFamide‐positive neurons forming the wall of the bladder (see [Fröbius and Funch 2017], Supporting Information Figure 6a, labelled “germovitellarial nerve loop”). Labelling of this area remained after hatching and was still detectable in the adult stages of *B. manjavacas*.

## 4 Discussion

Based on the high amino acid similarity, including the presence of common motifs in *ParaHox* and some of the *Hox* genes, both gene families are widely assumed to originate from a single ancient gene cluster predating the Cambrian explosion, thus reflecting genome duplication in early animal evolution (Brooke et al. 1998; Garcia‐Fernàndez 2005). The clustered arrangement of *Hox* genes is assumed to facilitate coordinated regulation and expression of these genes, allowing for the precise specification of regional identity along the anteroposterior axis (Duboule 1994). It is also believed to have provided a regulatory framework laying the grounds for diversifying body plans and evolving complex anatomical structures in bilaterian animals (Gellon and McGinnis 1998). Remnants of the ancestral arrangement of *ParaHox* genes can be observed in the genomes of several diploblastic animals (Hui et al. 2008; Steinworth et al. 2023). In addition, recent genomic data demonstrate that complete *ParaHox* gene complements arranged in a genomic cluster are present in some cnidarians (Law et al. 2023; Nong et al. 2020), consistent with *ParaHox* clustering predating the cnidarian‐bilaterian split. Within Bilateria, *ParaHox* gene clustering is particularly well conserved in deuterostome lineages (Furlong and Mulley 2008; Ikuta et al. 2013), where *ParaHox* genes are often retained in close proximity on the same chromosome.

The complement and genomic organisation of *ParaHox* genes, however, vary significantly between and even within the major protostome clades. The greatest variability of genomic organisation of *ParaHox* genes has been observed in the diverse Lophotrochozoa. With only a few exceptions—*Xlox* is missing in nemerteans (Luo et al. 2018) and no *Gsx* ortholog has been determined in platyhelminths so far (Saló et al. 2001)—all three *ParaHox* genes are found throughout lophotrochozoans (Biscotti et al. 2014; Ferrier and Holland 2001; Fritsch et al. 2016; Fröbius and Seaver 2006; Luo et al. 2018; Luo et al. 2015; Wang et al. 2017). While *ParaHox* gene clustering is retained within some lophotrochozoan groups (Wang et al. 2017), the genes occupy distant positions in most groups, even residing on different chromosomes. Likewise, Ecdysozoa are known for dispersed *ParaHox* clusters in addition to gene loss (Chipman et al. 2014; Kao et al. 2016; Wenyan Nong et al. 2020; Weiss et al. 1998). In particular, *Xlox* seems to have been lost early in ecdysozoan evolution. Within Ecdysozoa, *Gsx* has not been identified in nematodes (Ruvkun and Hobert 1998) and tardigrades (Boothby et al. 2015; Smith et al. 2016), consistent with lineage‐specific loss in these groups, whereas *Gsx* is present in priapulids.

Intriguingly, the *ParaHox* gene complements of Gnathifera mirror the ecdysozoan pattern. Our analysis suggests that *Xlox/Pdx* is absent from all gnathiferan genomes analyzed with subsequent loss of *Gsx* in some lineages (Mauer et al. 2021). Importantly, the lack of evidence for *Xlox* maintenance in Gnathifera is unlikely to reflect a technical artefact or assembly gap. Rather, *Xlox* absence is supported by extensive similarity searches across publicly available gnathiferan genomes and by independent validation using multiple genome assemblies. Irrespectively, the cluster is either dispersed or the genes retained are separated by several megabases in the gnathiferans studied. In addition, spatiotemporal expression of *ParaHox* genes in protostome development has yet attracted little attention, compared to their roles in deuterostome ontogenesis. In fact, our findings on spatiotemporal *ParaHox* expression in the monogonont rotifer *B. manjavacas* are novel for Gnathifera, and provide a first descriptive framework for *ParaHox* gene deployment in this lineage.

### 4.1 *Gsx* Patterns Parts of the Nervous System in Monogonont Rotifers

According to a long‐standing hypothesis, the digestive system of the last shared bilaterian ancestor was patterned collinearly to the structure of the *ParaHox* cluster as observed for *Hox* gene expression (Holland 2001). In the present study, however, we show that during embryogenesis of *B. manjavacas*, *Bm*‐*Gsx* transcripts are detected in two spatially restricted territories corresponding to parts of the nervous system. Diffuse posterior expression soon resolves into two cells in the foot region corresponding to FMRFamide‐positive neurons known to be part of the central nervous system (Fröbius and Funch 2017), connecting the foot to the caudal ganglion. In addition, smaller lateral expression domains in the midbody highlight two cells displaying a neural morphology, later becoming associated with the mastax. Under the experimental conditions used, no *Bm‐Gsx* expression was detected in the digestive tract of this gnathiferan representative, including the mouth, buccal tube, mastax, stomach, gastric glands, intestine, and cloaca. These observations are consistent with an involvement of *Gsx* genes in neural patterning in multiple metazoan clades (see below).

The *ParaHox* gene of the placozoan *Trichoplax adhaerens*, *Trox‐2*, is broadly expressed in the inner region of the lower epithelial layer along with *Elav*, a neural marker known from the anthozoan *Nematostella vectensis* (DuBuc et al. 2019). Furthermore, the lower epithelium of *T. adhaerens* is rich in neuropeptide‐secreting cells, with FFNPamide and YYamide expressing cells located in the region also expressing *Trox‐2* (Varoqueaux et al. 2018). Notably, the lower epithelium might be capable of sensing biofilms, which *T. adhaerens* takes up by phagocytosis (DuBuc et al. 2019). This could indicate that *Trox‐2* functions in the maintenance or differentiation of neurosensory cells, although the function of *Trox‐2* in placozoans remains to be clarified. If so, it would be consistent with data from the cnidarian *Hydra vulgaris*, where formation and regeneration of the apical nerve net near the hypostome, the oral region of the polyp, depends on *Cnox‐2/Gsx* expression in neuronal progenitors (Miljkovic‐Licina et al. 2007) and *Clytia hemispherica*, displaying *Gsx* expression in neural cells in tentacle bulbs of medusae (Chiori et al. 2009). However, comparisons across cnidarian lineages indicate considerable diversity in gene expression patterns along the oral‐aboral axis and throughout the life cycle (Chiori et al. 2009).

The involvement of *ParaHox* genes in neural patterning has been observed in other metazoan clades. For example, the anterior *ParaHox* gene *Gsx* exhibits expression predominantly in neural tissues in Deuterostomia. In echinoderms, *Gsx* is expressed in neuroectodermal domains (Arnone et al. 2006). *Gsx/Gsh* expression has also been reported from all higher‐ranking chordate clades. In the cephalochordate *B. floridae* and the urochordate *C. intestinalis, Gsx* is expressed in the anterior central nervous system (Brooke et al. 1998; Hudson and Lemaire 2001; Osborne et al. 2009). In vertebrates, distinct expression domains in varying parts of the neural tube and rhombomeres of the forming vertebrate brain have been observed (Cheesman and Eisen 2004; Deschet et al. 1998; Hsieh‐Li et al. 1995; Illes et al. 2009; Szucsik et al. 1997; Valerius et al. 1995). The pattern in Ecysozoa appears to be quite similar. For instance, the *Gsx* ortholog *Ind* (*intermediate neuroblast defective*) is upregulated in intermediate column cells of the developing central nervous system and a subset of brain neuroblasts in insects (Urbach and Technau 2003; Weiss et al. 1998; Wheeler et al. 2005).

*Gsx* expression in several lophotrochozoans is reminiscent of corresponding patterns in deuterostomes. For instance, *Gsx* is expressed in the anterior neuroectoderm during brain formation of direct‐developing annelids (Fröbius and Seaver 2006) and in cerebral, optic and palliovisceral ganglia in direct‐developing cephalopods (Wollesen et al. 2015). Only lophotrochozoans with indirect development have additional expression domains in the apical organ, a sensory organ in the episphere of the trochophore and various structures of the digestive tract (stomodeum, midgut, digestive gland, foregut) (Hui et al. 2009; Kulakova et al. 2008; Samadi and Steiner 2010)). Thus, *Gsx* genes may have acquired additional functions in these lineages. In contrast to the latter, *Gsx/Ind* expression is absent in the digestive tract of all ecdysozoan species studied so far (Urbach and Technau 2003; Weiss et al. 1998; Wheeler et al. 2005).

As outlined above, *Gsx* expression patterns differ between animals. Nevertheless, an involvement in neuronal patterning as observed by us in the gnathiferan *B. manjavacas* is widespread in animals, and might have emerged in early metazoan evolution.

### 4.2 Rotiferan *Cdx* Expression Is Restricted to Neuroepithelial Cells Forming the Bladder

In rotifers, *Cdx* expression exhibits a distinctive and highly restricted pattern. In *B. manjavacas*, in situ hybridization showed that *Bm‐Cdx* transcripts first appear in cells forming the osmotic vesicle (bladder), a pulsating organ connected to the excretory system and located in the posterior trunk region (Fontaneto and De Smet. 2015). Observations suggest that this organ derives from posterior ectoderm, as the initially labelled cells originate from the surface of the posterior embryonic pole. Interestingly, *Bm‐Cdx*‐positive bladder cells are not solely epithelial: they display features consistent with neural identity, including immunoreactivity for the neurotransmitter FMRFamide and apparent connections to the central nervous system. Taken together, these observations suggest that, in rotifers, *Cdx* expression does not pattern the digestive tract but rather ectodermal bladder cells exhibiting neural characteristics.

Previous analyses have shown that several rotifer *Hox* genes are expressed in posterior neural structures rather than being associated with discrete morphological subdivisions. In *B. manjavacas*, *Hox3* is expressed in the caudal ganglion and in neurons innervating the pedal musculature and the foot, *Hox4* in caudal nerves innervating the foot, and *Hox6* in the caudal ganglion with a possible neural connection to the germovitellarium, while *MedPost* is restricted to a small number of cells in the foot and near or within the caudal ganglion (Fröbius and Funch 2017). In addition, the anterior *Hox* gene *Hox2* is expressed in neural elements associated with the stomatogastric nervous system. Importantly, the rotifer foot does not represent an appendage but rather a postanal tail‐like structure. These *Hox* expression patterns suggest that anteroposterior patterning information in rotifers is prominently deployed within functionally specialized neural circuits.

The *Hox* gene expression patterns described above resemble those we report here for *ParaHox*. Like *Hox3*, *Gsx* is expressed in cells corresponding to connections between the foot and the caudal ganglion and in cells associated with the stomatogastric nervous system, whereas *Cdx* is detected in neuroepithelial cells of the posterior excretory system similar to *Hox2*. Notably, *ParaHox* expression was not detected in the digestive tract or in the brain under the experimental conditions used, and rotifers lack both *Hox1* and the posterior‐most *Hox*/*Post* genes present in many other bilaterians. Together, these findings foster the interpretation that, in Rotifera, *Hox* and *ParaHox* genes are deployed within neural patterning systems that overlap with, and complement, *Hox* gene expression. We interpret this condition as a lineage‐specific deployment of *ParaHox* function, potentially involving partial or complete reduction of non‐neural gut expression, which refines but does not contradict hypotheses of *ParaHox* roles in both nervous system and gut patterning in bilaterians. This finding is notable when compared to the broader diversity of *Cdx* expression patterns across animals. In most bilaterians studied to date, *Cdx* expression occurs in posterior domains and involves different germ layers and their derivatives, such as the hindgut (endodermal or ectodermal), posterior mesoderm, or parts of the developing nervous system, as summarized below. The rotifer pattern, confined to a neuroectodermal derivative of the excretory system, therefore contrasts with more widespread posterior digestive and mesodermal associations reported in other groups.

The presence of *Cdx* orthologs outside Bilateria has been debated. In particular, genomic searches have failed to identify *Cdx* genes in placozoans and ctenophores, and results for Porifera have been inconsistent. While some analyses did not recover *Cdx* genes in sponges, other studies identified a *Cdx*‐like gene in calcisponges and demonstrated its expression in oocytes and in the inner cell mass during choanocyte chamber formation (Fortunato et al. 2014). Given proposed homologies between the sponge choanoderm and bilaterian endoderm (Leininger et al. 2014), these findings align with an early association of *Cdx*‐related genes with endodermal or gut‐related tissues. Accordingly, the presence of *Cdx* in Porifera should be regarded as an open question rather than a resolved issue. However, in cnidarians, a gene with similarity to both *Cdx* and *Xlox* has been identified (Hui et al. 2008). In *N. vectensis*, this gene (NVHD065) is expressed in restricted endodermal domains, specifically flanking the ventral midline and at the tentacle basis (Ryan et al. 2007). Thus, even in non‐bilaterian animals, expression associates with localized endodermal regions, which may reflect maintenance of ancestral *Cdx/Xlox*‐related gene functions in these taxa. Either way, representatives of Xenacoelomorpha, which includes acoels, nemertodermatids, and xenoturbellids, clearly have *Cdx* orthologs. Expression analysis in the acoel *Convolutriloba longifissura* revealed two domains: one within the anterior central nervous system and another in posterior ectoderm, in the region of the future gonopore (Hejnol and Martindale 2008, 2009). This pattern, combining neural and posterior ectodermal expression, bears similarity to the rotifer case, where neural characteristics coincide with posterior ectodermal derivatives.

Further details on *Cdx* gene function come from deuterostomes, where expression has been documented in all major groups. For example, *Cdx* expression localizes to the posterior endoderm of the archenteron in echinoderms (*Strongylocentrotus purpuratus*, *Patiria miniata*) and hemichordates (*Ptychodera flava*) (Annunziata et al. 2013; Arnone et al. 2006; Ikuta et al. 2013). Expression in neuroectoderm, however, has been reported only from chordates. In ascidians (e.g. *Halocynthia roretzi*, *C. intestinalis*), *Cdx* expression is detected in the posterior neural tube and tail during embryonic and larval development, whereas gut expression becomes apparent only after metamorphosis (Imai et al. 2009; Katsuyama et al. 1999; Nakayama et al. 2016). In cephalochordates (*Branchiostoma*), expression occurs both in the posterior neural tube and in gut tissue, contributing to body axis extension and anus formation (Brooke et al. 1998; Zhong et al. 2020). In vertebrates, multiple *Cdx* paralogs arose along with whole‐genome duplications (Marlétaz et al. 2015). These paralogs have been implicated in gut development, posterior identity along the axis, and regulation of *Hox* genes in the paraxial mesoderm (Kumar et al. 2003; Marlétaz et al. 2015; van den Akker et al. 2002). Together, deuterostome data highlight dual involvement of *Cdx* in gut and neural tissues, with prominent roles in posterior axis formation.

In ecdysozoans, *Cdx* orthologs acquired additional functions beyond their primary role in posterior patterning. In *Caenorhabditis elegans*, the ortholog *pal‐1* specifies posterior hypodermis, muscle, and rectum through transactivation of the *Hox* gene *mab‐5*, and postembryonically regulates neurogenesis of male tail sensory rays (Edgar et al. 2001; Waring and Kenyon 1991). In priapulids, expression occurs at the vegetal pole of the gastrula and in posterior ectoderm at the establishment of the boundary between the trunk and anterior proboscis, with an additional possible neurogenic domain in the proboscis (Martín‐Durán et al. 2012). Arthropods have been studied extensively: the ortholog *Caudal* (*Cad*) contributes to posterior body patterning in short‐ and intermediate‐germband insects (Dearden and Akam 2001; Schulz et al. 1998). It might further be involved in gut formation in representatives like *Drosophila* (Wu and Lengyel 1998). However, such a role most likely reflects a complementary involvement of *Cad* in long‐germband insects as the mode of short‐ or intermediate‐germband segmentation is regarded to be ancestral (Davis and Patel 2002). Thus, ecdysozoan *caudal* genes are generally involved in posterior specification, with additional roles in neurogenesis or gut development depending on lineage.

In lophotrochozoans, *Cdx* expression is strongly tied to hindgut development. This is documented in brachiopods and phoronids (Andrikou et al. 2019), ectoprocts (Vellutini et al. 2017), molluscs (Johnson and Lambert 2020), and polychaetes, with expression consistently being localized to posterior gut regions (Fröbius and Seaver 2006; Hui et al. 2009; Kulakova et al. 2008). In nemerteans, *Cdx* expression spans both ectodermal and endodermal hindgut derivatives (Hiebert and Maslakova 2015; Martín‐Durán et al. 2015). These observations indicate that hindgut expression of *Cdx* is broadly conserved across lophotrochozoans, although gene loss might sporadically have occurred. In fact, flatworms lack *Cdx* genes altogether and do not form a hindgut either (Martín‐Durán and Romero 2011).

Expression of rotifer *Cdx*, characterized by a posterior expression domain with neural features, differs from patterns in deuterostomes, ecdysozoans and lophotrochozoans as detailed above. This variation in *Cdx* expression across metazoans highlights a complex evolutionary history of *Cdx* gene deployment.

## 5 Conclusions

The original hypothesis for the ancestral function of *ParaHox* genes in the last common bilaterian ancestor emphasized gut patterning. Following the principle of spatial collinearity with the genomic *ParaHox* cluster structure, it had been proposed that the “anterior” *ParaHox* gene *Gsx* patterns the foregut, the “middle” gene *Xlox* the midgut and the “posterior” gene *Cdx* the posterior part of the digestive tract. Meanwhile, however, both genomic organisation and expression of this gene family have been analysed not only in a variety of ecdysozoan, lophotrochozoan and additional deuterostomian clades but also in non‐bilaterian body plans. Intact *ParaHox* clusters seem to be the exception rather than the rule, excluding collinearity principles for many taxa.

Comparative data indicate that *ParaHox* genes can be involved in both the regionalization of the gut along the anteroposterior axis and the patterning of the nervous system, although the relative contributions of these roles vary among lineages. The results of our examination of gnathiferan *ParaHox* gene expression, however, suggest that, in rotifers, these two functions may not be strictly separable. *Bm‐Gsx* is expressed in a spatially restricted domain corresponding to the stomatogastric nervous system, and *Bm‐Cdx* is expressed in epithelial cells of the osmotic vesicle that display neural features, consistent with an association with posterior neural circuitry. Likewise, the animal gut is not just a simple pipe derived from a single germ layer but consists of different cell types (Noah et al. 2011). Epithelial cells exhibiting neural or sensory functions are well‐documented across Platyhelminthes, including free‐living planarians and related rhabditophorans, as well as in nematodes (Collins 2017; Gąsiorowski et al. 2023; Webb and Davey 1975). During gut formation, the integration of sensory cells and other neurons ensures the correct function of all processes. Given the extremely miniaturized body plan and eutely of rotifers, with fixed cell numbers and constrained developmental trajectories, the *ParaHox* gene expression patterns observed here are best interpreted as a lineage‐specific deployment shaped by these developmental constraints.

## Author Contributions

Andreas C. Fröbius conceived and designed the experiments and performed the bulk of the acquisition, analysis, and interpretation of the data. David B. Mark Welch and Holger Herlyn provided genomic data. Figures were generated, and the manuscript was drafted by Andreas C. Fröbius. Andreas C. Fröbius, Holger Herlyn and David B. Mark Welch made substantial revisions to the manuscript and approved the article.

## Funding

The authors received no specific funding for this work.

## Ethics Statement

The authors have nothing to report.

## Consent

The authors have nothing to report.

## Conflicts of Interest

The authors declare no conflicts of interest.

## Supporting information


**Table S1:** Taxa, species, and accession numbers of sequences used in phylogenetic analyses.

Figure_S1.

## Data Availability

New sequence data from this study were submitted to GenBank at NCBI under accession numbers PV294965 and PV294966. Additional sequences used for phylogenetic analyses were obtained from GenBank; accession numbers are available within the paper. Genomic datasets used are also available at NCBI (https://www.ncbi.nlm.nih.gov/datasets/genome/).
