# Supplementary material for: ParaHox Genes Revisited: From Gut Patterning to Integrated Axial and Neural Organization in Rotifera
Source: J Exp Zool B Mol Dev Evol. 2026 Mar 15;346(4):357–69. doi: 10.1002/jez.b.70019 (PMC13161344; doi:10.1002/jez.b.70019)
Supplement: Supplementary file 1 — Table S1: Taxa, species, and accession numbers of sequences used in phylogenetic analyses. [file JEZ-346-357-s001.docx]

**Table S1 Taxa, species, and accession numbers of sequences used in phylogenetic analyses**

| **Taxon** | **Art** | **Accession number(s)** |
| --- | --- | --- |
| **Placozoa** | *Trichoplax adhaerens* | AAQ82695.1 |
| **Cnidaria** | *Hydra vulgaris* | AKQ09536.1 |
| **Cnidaria** | *Podocoryne carnea* | AAG09805.1 |
| **Cnidaria** | *Clytia hemisphaerica* | ACM62729.1 |
| **Xenacoelomorpha** | *Symsagittifera roscoffensis* | AFD32742.1; AAN11406.1; ACM69150.1 |
| **Xenacoelomorpha** | *Convolutriloba longifissura* | ACG70807.1 |
| **Xenacoelomorpha** | *Hofstenia miamia* | QFQ66879.1 |
| **Gnathifera – Bdelloidea** | *Adineta vaga* | genome-derived |
| **Gnathifera – Acanthocephala** | *Pomphorhynchus laevis* | genome/transcriptome-derived |
| **Gnathifera – Chaetognatha** | *Flaccisagitta enflata* | ABS18808.1; ABS18809.1; ABS18815.1; ABS18816.1 |
| **Annelida** | *Platynereis dumerilii* | ACH87540.1; ACH87546.1; AFJ91921.1 |
| **Annelida** | *Chaetopterus variegatus* | AAD55932.1 |
| **Annelida** | *Capitella teleta* | ABY67961.1; ABY67960.1 |
| **Nemertini** | *Lineus sanguineus* | P81193.1 |
| **Nemertini** | *Maculaura alaskensis* | AKE07576.1; AKE07580.1; AKE07584.1 |
| **Mollusca** | *Acanthochitona crinita* | AMT82760.1; APD15641.1 |
| **Mollusca** | *Euprymna scolopes* | AAV85466.1; AAL25811.1; AAL25812.1 |
| **Bryozoa** | *Bugula neritina* | AEH57082.1 |
| **Brachiopoda** | *Terebratalia transversa* | AHY88468.1; ANO46569.1 |
| **Brachiopoda** | *Lingula anatina* | AAD45594.1; AAD45595.1 |
| **Entoprocta** | *Pedicellina cernua* | ALQ28251.1 |
| **Priapulida** | *Priapulus caudatus* | AFY12010.1; XP_014665932.1 |
| **Tardigrada** | *Hypsibius dujardini* | ALT32058.1 |
| **Tardigrada** | *Ramazzottius varieornatus* | GAU91984.1 |
| **Onychophora** | *Euperipatoides kanangrensis* | CEP25538.1; CCK73379.1; CCK73369.1 |
| **Crustacea** | *Armadillidium nasatum* | KAB7499970.1 |
| **Crustacea** | *Daphnia pulex* | EFX86750.1 |
| **Chelicerata** | *Parasteatoda tepidariorum* | LAA04824.1 |
| **Chelicerata** | *Archegozetes longisetosus* | AGV52782.1 |
| **Chelicerata** | *Cupiennius salei* | CAB40807.1 |
| **Insecta** | *Ceratina calcarata* | LAA04824.1 |
| **Insecta** | *Drosophila melanogaster* | NP_996087.2; ABS01374.1 |
| **Insecta** | *Tribolium castaneum* | AAW21974.1; NP_001034498.1; NP_001107762.1 |
| **Insecta** | *Temnothorax longispinosus* | TGZ52842.1 |
| **Myriapoda** | *Strigamia maritima* | AAT35589.1 |
| **Hemichordata** | *Saccoglossus kowalevskii* | NP_001158415.1; AAP79296.1; NP_001158381.1; NP_001158414.1; NP_001158413.1; NP_001158412.1 |
| **Hemichordata** | *Ptychodera flava* | AAR07642.1; AAR07645.1; AAR07634.1 |
| **Echinodermata** | *Patiria miniata* | AGK89736.1; AGK89735.1 |
| **Echinodermata** | *Metacrinus rotundus* | BAF43721.1 |
| **Cephalochordata** | *Branchiostoma lanceolatum* | ACJ74382.1; ACJ74386.1; ACJ74389.1; ACJ74391.1; ACJ74390.1; ACJ74393.1 |
| **Cephalochordata** | *Branchiostoma floridae* | AAF81909.1 |
| **Urochordata** | *Herdmania curvata* | AAF60348.1 |
| **Urochordata** | *Ciona intestinalis* | NP_001027663.1 |
| **Urochordata** | *Oikopleura dioica* | AAS21474.1 |
| **Urochordata** | *Diplosoma listerianum* | AAP47021.1 |
| **Vertebrata** | *Mus musculus* | NP_032204.1; NP_034010.3 |
