# Supplementary figures and images for: ParaHox Genes Revisited: From Gut Patterning to Integrated Axial and Neural Organization in Rotifera
Source: J Exp Zool B Mol Dev Evol. 2026 Mar 15;346(4):357–69. doi: 10.1002/jez.b.70019 (PMC13161344; doi:10.1002/jez.b.70019)

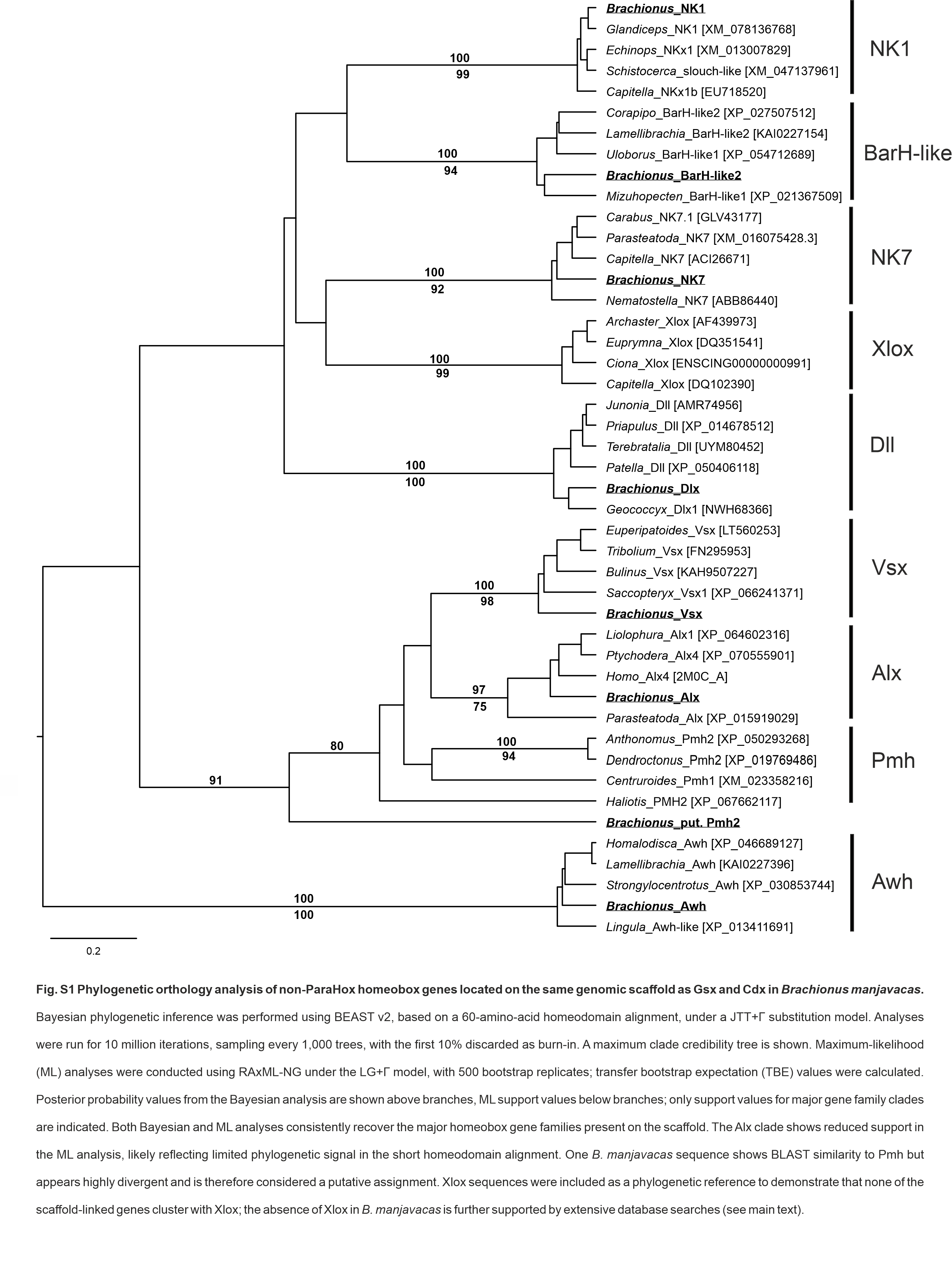

Supplement: Supplementary file 2 — Figure_S1. [file JEZ-346-357-s002.png]
